# Prognostic Value of Focal Adhesion Kinase (FAK) in Human Solid Carcinomas: A Meta-Analysis

**DOI:** 10.1371/journal.pone.0162666

**Published:** 2016-09-16

**Authors:** Xiao-Qing Zeng, Na Li, Li-Li Ma, Yu-Jen Tseng, Nai-Qing Zhao, Shi-Yao Chen

**Affiliations:** 1 Department of Gastroenterology, Zhongshan Hospital, Fudan University, Shanghai, China; 2 Endoscopy Center and Endoscopy Research Institute, Zhongshan Hospital, Fudan University, Shanghai, China; 3 Department of Biostatistics, Fudan University, Shanghai, China; 4 Evidence-based Medicine Center, Fudan University, Shanghai, China; Academia Sinica, TAIWAN

## Abstract

**Background:**

Recently, the number of reports on focal adhesion kinase (FAK) as a vital therapeutic target in solid carcinomas has increased; however, the prognostic role of FAK status remains poorly understood. This study aims to evaluate the prognostic effect of FAK by means of a meta-analysis.

**Methods:**

We performed a systematic literature search in order to examine the correlation between expression of FAK and overall survival(OS). The hazard ratio (HR) of OS was used to measure survival. A random-effects model was used to pool study statistics. Sensitivity and publication bias analyses were also conducted.

**Results:**

Thirty eligible studies involving 4702 patients were included. The median expression rate of FAK was 54%. Meta-analysis of the HRs demonstrated that high FAK expression was associated with worse OS (average HR = 2.073, 95%confidence interval[CI]:1.712–2.510, p = 0.000). Regarding cancer type, FAK was associated with worse OS in gastric cancer (HR = 2.646,95% CI:1.743–4.017, p = 0.000), hepatocellular carcinoma (HR = 1.788,95% CI:1.228–2.602, p = 0.002), ovarian cancer (HR = 1.815, 95% CI: 1.193–2.762, p = 0.005), endometrial cancer (HR = 4.149, 95% CI:2.832–6.079, p = 0.000), gliomas (HR = 2.650, 95% CI: 1.205–5.829, p = 0.015), and squamous cell carcinoma (HR = 1,696, 95% CI: 1.030–2.793, p = 0.038). No association was found between HR and disease staging according to our meta-regression analysis.

**Conclusions:**

Our study shows that high expression of FAK is associated with a worse OS in patients with carcinomas, but the association between FAK and prognosis varies according to cancer type. The value of FAK status in clinical prognosis in cancer needs further research.

## Introduction

Prognostic studies of cancer biomarkers are valuable, and allow a more accurate prediction of treatment response and prognosis, ultimately leading to a favorable therapeutic outcome. Focal adhesion kinase (FAK), an intracellular tyrosine kinase recruited to sites of integrin clustering or focal adhesions, is a multi-functional regulator of cell signaling within the tumor microenvironment [1–3]. FAK functions as a major mediator of signal transduction by cell surface receptors including integrins, growth factor, and cytokine receptors [1]. Therefore, FAK plays a crucial role in tumor carcinogenesis, especially in cell proliferation, apoptosis inhibition, angiogenesis, invasiveness, immunosuppression, and cell motility [4]. Dysregulation of FAK leads to the development of malignancies, including initiation of invasion, metastasis and neoangiogenesis [5–7]. These functional characteristics suggest that FAK may be also involved in promoting tumorigenesis and metastasis.

Recently, FAK has been proposed as a new candidate for molecular-based therapeutic approaches. However, the prognostic value of FAK overexpression across human solid carcinomas has yet to achieve a recognized consensus. Controversial results have been reported among the different types of cancer. High FAK expression in cancer samples has been evident in hepatocellular carcinoma [8], invasive breast carcinoma [9], gastric carcinoma [10], endometrial cancer [11], and ovarian carcinoma [12]. It has been demonstrated that the overexpression of FAK in these carcinomas is associated with a worse outcome. On the other hand, there are conflicting data demonstrating no prognostic value of FAK expression in node-negative breast cancer, colon carcinoma, and resectable pancreatic cancer[13–15]. In addition, overexpressed FAK was linked with poorer survival rates in esophageal and head and neck squamous cell carcinoma patients, although no statistical significance was established[16,17]. Thus, a systematic and comprehensive meta-analysis designed to explore the association of FAK overexpression with cancer prognosis is urgently required and will provide a useful reference for doctors and researchers working in this field.

## Materials and Methods

### Literature search

A search of Pubmed and Web of Science was performed for studies evaluating the expression of FAK and survival in tumors from January 1995 to April 2016. The following keywords were used in the search strategy: (FAK OR “focal adhesi* kinase”) AND (Carcinoma* OR neoplasm* OR cancer OR tumor) AND (prognosis OR prognostic OR survival). The results were limited to English language studies. Manual searches of reference articles from applicable studies were performed to identify articles that may have been missed by the computer-assisted search.

### Study selection

Two investigators (Zeng and Li) reviewed all citation titles identified by the search strategy to generate a list of potentially relevant articles. The abstract of each study was then reviewed individually by the two investigators. If the applicability of a study could not be determined through the title or abstract alone, the full text was reviewed. The articles were independently screened for possible eligibility and any disagreements were resolved by conferring with a third investigator (Chen).

### Study inclusion and exclusion criteria

The meta-analysis included studies that met the following standards: i) all patients were diagnosed with cancer via histopathology; ii) reported FAK expression level in patients and their prognoses; iii) original study; iv)inclusion of the most complete and newest study if duplicate articles were published; v) reported explicit methods for the detection of FAK expression in solid cancers; vi) FAK data presented in dichotomy; high or low. The exclusion criteria were as follows: i)studies investigating the relationship between co-expression of FAK and other factors and prognosis; ii) studies with no hazard ratios (HR) or 95% confidence intervals (CI), or data failed to provide a Kaplan-Meier(K-M) curve for HR and CI calculations; iii) abstracts were excluded due to insufficient data to evaluate the methodological quality of the trial and/or to carry out a meta-analysis; iv) non-eligible trials included ecological studies, case reports, reviews, editorials, and animal trials. If a study reported results from more than one method (i.e., immunohisto-chemistry [IHC], polymerase chain reaction[PCR] and fluorescence *in situ* hybridization[FISH]), for more than one well described patient group or with multiple cut off values, results of all analyses were included in the meta-analysis. This study was conducted in accordance with the PRISMA guidelines [18].

### Data extraction

The following characteristics were extracted: name of the first author, publication year, country, sample size, age, sex, test method, cut-off value, tumor staging, follow up time, and HR estimation. If a study reported both the results of univariate and multivariate analysis, the latter was selected as it takes confounding factors into account. The primary outcome measure was overall survival (OS).

### Quality assessment

For study methodological evaluation, three investigators (Zeng, Li, and Chen) read through each publication independently. There is no widely accepted standard for evaluating study quality, thus study quality was assessed and scored according to the REMARK guidelines and quality scale predefined form by De Graeff [19,20], which was adapted from McShane et al. (2005) and Hayes et al. (1996). Studies with a total score of 8 were considered to show optimal study quality, whereas a score of 0 indicated poor study quality. The three readers provided independent quality scores for comparison, then a mutual consensus was reached for each item.

### Statistical analysis

The expression of FAK was judged “high” or “low” according to the cut-off value used in each study. The association between FAK and clinical outcomes was evaluated using the HR of low FAK expression level patients over high FAK level patients.

When described in original articles, HR values were obtained directly. If the data were not provided directly, the available data from K-M survival curves were interpreted through Getdata. Three independent authors read the curves to minimize reading variability. HR and 95% CI were calculated using the methods reported by Parmar [21].

Estimates of HRs were weighted and pooled using the Mantel-Haenszel random-effects model. The heterogeneity of results between studies was assessed using *I*^*2*^ statistics, with increasing heterogeneity implying less utility in generalization across studies. The χ^2^-test P-value<0.10 or *I*^*2*^ values >50% were suggestive of substantial heterogeneity. A sensitivity analysis was conducted to evaluate sources of heterogeneity both in the overall pooled estimate as well as within subgroups. Subgroup analysis was investigated with respect to cancer, ethnicity, assay method, HR estimate, type of tumor, sample size, and study quality score. Meta-regression analysis was conducted in an attempt to establish the relationship between HR and disease stage. To test the robustness of the HR estimates, a sensitivity analysis was conducted by individually excluding studies and analyzing the effects on the remaining studies. In addition, the presence of publication bias which was assessed using the Begg’s test. All analyses were carried out using Stata 12.0. All P-values were two-sided and the significance level was defined as 0.05.

## Results

### Literature search

**Fig 1:**
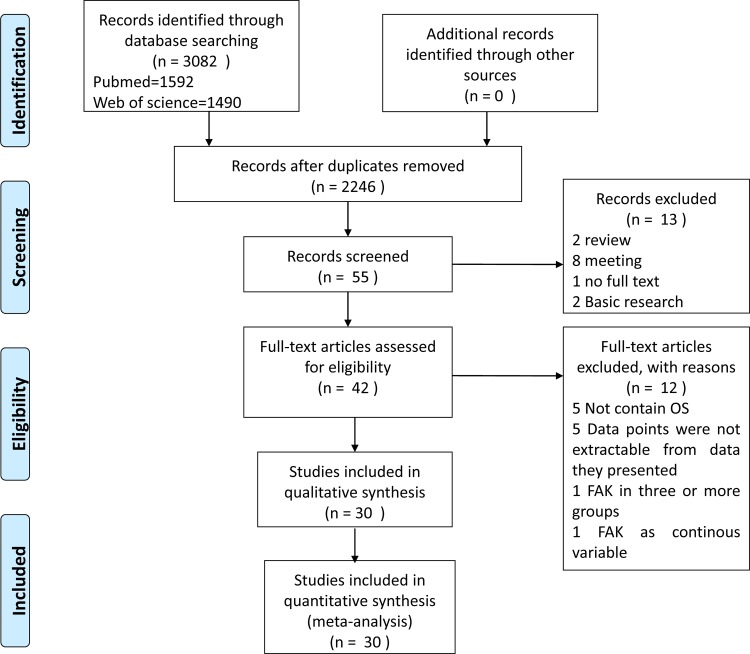
Selection of studies included in the analysis.

The computer-assisted search yielded 2,246 unique published titles. After an initial review, 55 titles were considered to be potentially appropriate. The abstracts of each of these papers was reviewed, after which 13 studies were excluded. The full text was not available for one article [22]. The full text of the remaining 42 citations was then carefully read, and twelve articles were subsequently excluded. Of the excluded articles, five employed an alternative survival endpoint instead of OS [23–27], five had no data available[8,13–14,28–30], one divided the FAK level into three or more groups [30], and one article treated FAK scores as a continuous variable[31]. A total of 30 studies were thus ultimately included in our meta-analysis (Fig 1) [10,15,16,32–58].

### Characteristics of included studies

The principal characteristics of the selected studies are summarized in Table 1. From the 30 studies included, a total of 4702 patients were analyzed. Chen et al.[33] stratified the data into a training and validation set, presenting the two groups separately. The outcome data of two studies were presented independently according to the method of ascertaining FAK expression in the same patient population, and these datasets were analyzed separately. Of these, Park et al. [47] evaluated FAK protein expression in both the cytoplasm and the cell membrane, and the datasets were interpreted individually. Three studies presented FAK expression distinctly according FAK status and these datasets were treated separately [32,41,43]. Specifically, Giaginis et al. [10] stratified outcome data into two histological types of human gastric cancer, of which the subgroup of diffuse-type carcinomas was excluded since the relevant effect estimates could not be obtained.

**Table 1:** Characteristics of studies reporting FAK expression and outcomes.

Ref	Type of cancer	country	Age, median(range)	Male/fe-male	advanced stage(%)	FAK+ tumor(%)	HR(95%CI)
Albasri 2014	CRC	U.K	72(45–80)	257/192	43.3%	44.0%	1.129(0.763–2.12)
Dy 2014	NSCLC	USA	68(46–86)	75/82	0%	56.7%	0.79(0.53–1.16) *
Zhang 2014	GC	China	64(30–91)	428/173	56.4%	54.1%	2.463(1.119–5.405)
Gao 2014	HCC	China	NR	146/14	NR	51.3%	1.613(1.035–2.514)
Chen 2013(training)	GC	China	NR	61/21	67.1%	52.4%	7.76(3.76–16.0)
Chen 2013(validation)	GC	China	NR	296/89	51.7%	64.7%	1.70(1.23–2.34)
Ji 2013	NSCLC	China	58.1#(31–78)	97/56	42.6%	38.6%	2.22 (1.43–3.44) *
Zhou 2013(FAK)	Endometrial carcinoma	USA	63.2#(24–91)	0/202	42.6%	46.0%	3.79(2.01–7.13) *
Zhou 2013(pFAK)	Endometrial carcinoma	USA	63.2#(24–91)	0/202	42.6%	64.9%	4.37(2.71–7.06) *
Garouniatis 2013	CRC	Greece	69#(36–90)	96/87	38.8%	31.7%	2.517(1.468–4.315)
Kim 2012	CRC	Korea	62.2#(28–84)	136/84	48.0%	61.1%	1.382(0.813–2.349)
Qayyum 2012	Renal cancer	U.K	60(41–80)	32/25	36.8%	36.8%	3.35(1.40–7.98)
Theocharis 2012	Tonque SCC	Greece	60(33–94)	25/23	NR	50%	2.431(0.747–6.698)
Fan 2011	Ovarian cancer	China	NR	0/60	100%	73.3%	3.922(1.126–13.664)
Yom 2011(FISH)	Breast cancer	Korea	NR	0/240	36.3%	13.8%	5.393(2.185–13.308)
Yom 2011(IHC)	Breast cancer	Korea	NR	0/240	36.3%	29.2%	0.964(0.383–2.425)
Park 2010(IHC cyto)	GC	Korea	NR	322/122	36.5%	84.2%	4.57(2.20–9.48)
Park 2010(IHC mem)	GC	Korea	NR	322/122	36.5%	80.0%	3.17(1.73–5.80)
Park 2010(FISH)	GC	Korea	NR	281/103	32.8%	8.9%	1.71(1.32–2.23)
Yuan 2010	HCC	China	48.5#(13–72)	47/3	54.0%	50%	1.3(0.13–12.61) *
Chatzizacharias 2010	PDAC	Greece	NR	NR	27.7%	35.4%	0.97(0.59–1.59) *
Ding 2010(FAK)	Gliomas	China	NR	61/35	81.3%	89.6%	3.25(0.8–13.15) *
Ding 2010(pFAK)	Gliomas	China	NR	61/35	81.3%	50.0%	2.41(0.93–6.27) *
Hayashi 2010	EBD carcinoma	Japan	66.1#(NR)	54/22	18.4%	77.6%	2.239(0.894–5.603)
Giaginis 2009	GC	Greece	NR	22/8	63.3%	56.7%	0.93(0.2–4.32) *
Wang 2009	Lung adnocarcinoma	China	60.1#(NR)	42/35	34.3%	87.8%	3.28(1.335–8.064)
Sun 2007	HCC	China	NR	78/94	NR	57.1%	2.42(0.59–9.87)
Furuyama 2006	Pancreatic cancer	Japan	64.3#(45–79)	31/19	46%	48.0%	0.69 (0.18–7.28) *
Ohta 2006	ICC	Japan	60.3#(33–75)	38/18	83.9%	28.6%	1.62(0.47–5.52)
Sood 2004	Ovarian cancer	USA	59.3#(34–81)	0/79	81.0%	68.4%	1.93 (0.51–7.28) *
Itoh 2004	HCC	Japan	NR	47/17	23.4%	28.1%	3.05(1.16–7.99)
Miyazaki 2003	ESCC	Japan	61#(NR)	77/14	42.9%	59.3%	1.04 (0.17–6.19) *
Jan 2009	HCC	Taiwan	60(34–80)	39/16	42%	61.8%	1.02 (0.14–7.37) *
Li 2012	Laryngeal SCC	China	51(37–84)	81/5	73.3%	73.3%	1.611(0.84–2.73)
Aust 2014(FAK)	Ovarian cancer	Austria	57.6#	0/179	96%	92.2%	1.10(0.45–2.70)
Aust 2014(pFAK)	Ovarian cancer	Austria	57.6#	0/179	96%	36.9%	1.85(1.04–3.23)
Li 2015	Ovarian cancer	China	50.5#(NR)	0/50	83.9%	72.0%	2.52(0.12–52.68)*

CRC, colorectal cancer; NSCLC, non-small-cell lung cancer; GC, gastric cancer; HCC, hepatocellular carcinoma; OSCCs, oral squamous cell carcinomas; SCC, squamous cell carcinoma;SCLC, small-cell lung carcinoma; EBD, extrahepatic bile duct; ICC, intrahepatic cholangiocarcinoma; ESCC, oesophageal squamous cell carcinoma; PDAC, pancreatic ductal adenocarcinoma; cyto: cytoplasmic; mem: membranous; NR, not report; HR, hazard ratio; CI, confidence interval

# mean

*survival curve.

All of the studies were retrospective in design. Sample sizes ranged from 30 to 601 (median, 96). Eighteen of the 30 studies included patients with both early and advanced disease (stage I–IV) [10,15,16,33,34–38,44,46,47,51–54,56–58]. However, none of the studies analyzed the OS according to stage. Five studies included patients with stage I–III disease [32,40,42,43,48,55], two studies included patients with stage II–IV disease [41,43], and one study each included patients with stage I disease [45] and stage III–IV disease [49]. Stages of cancer were not reported in three studies [34,39,50]. The mean percentage of patients with advanced stages of disease was 52.0% (range, 0–100%). Five studies evaluated hepatocellular carcinoma [34,35,39,46,56], Four studies each evaluated gastric cancer [10,33,37,47] and ovarian cancer [36,41,49,58], three studies each evaluated lung cancer [40,45,55], and colorectal cancer[52–54], two studies each evaluated pancreatic cancer [15,44], and cholangiocarcinoma [38,42], and one study each evaluated breast cancer [48], gliomas [49], endometrial carcinoma [32], esophageal squamous cell carcinoma [16], renal cancer [51], tongue squamous cell carcinoma [50], and laryngeal squamous cell carcinoma [57]. Twenty studies were performed in Asian populations, while the remaining 10 studies were conducted in Western populations.

Follow-up time was 42 months (range, 0.1–192.2 months). Eighteen studies had readily available HR and 95% CI data, while the remaining 12 studies presented neither HR nor 95% CI, which were consequently estimated from the available K-M curves. The 30 included studies had a mean quality score of 3 (range, from 1–6).

### Evaluation and expression of FAK

The rate of high FAK status varied from 8.9–92.2% (median, 54%) (Table 1). The rate of high FAK expression was 57% (range, 28.1–92.2%) in studies using IHC and 14% (range, 8.9–51.3%) in studies using other methods. A description of the antibodies and cut off values of overexpression used is given in S1 Table. Various antibodies were used for the evaluation of FAK expression. Among the 34 datasets that employed IHC, the FAK expression level was evaluated according to the intensity of staining, percentage of stained cells and method applied. Marked heterogeneity was observed in studies evaluating FAK expression by IHC between thresholds used to dichotomize FAK status. Among the three datasets which assigned FAK expression at the gene level, FISH was used in two datasets[47,48], and PCR was used in the remaining one [34].

### Association of FAK with survival

**Fig 2:**
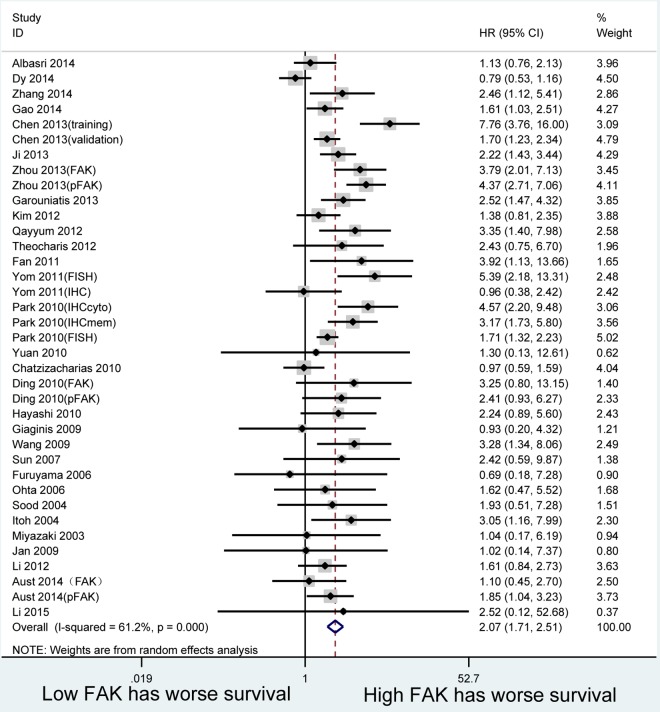
Forest plots. Hazard ratios for each study are represented by the squares, the size of square represents the weight of the study in the meta-analysis, and the horizontal line crossing the square represents the 95% confidence interval(CI). All statistical tests were two-sided.

The combined HR for the 30 studies included in the analysis was 2.073 (95% CI:1.712–2.510, p = 0.000), indicating that FAK overexpression is associated with worse survival among patients. However, a significant inter-study heterogeneity effect model (*I*^*2*^ = 61.2%, p = 0.000) was indicated for the prognostic effect (Fig 2).

To explore the study heterogeneity, we performed stratified analyses across a number of key study characteristics and clinical factors (Table 2). In the subgroup analysis based on tumor types, the negative prognostic role of high FAK expression was observed in gastric cancer (HR = 2.646, 95% CI:1.743–4.017, p = 0.000), hepatocellular carcinoma (HR = 1.788, 95% CI:1.228–2.602, p = 0.002), ovarian cancer (HR = 1.815, 95% CI: 1.193–2.762, p = 0.005), endometrial cancer (HR = 4.149, 95% CI: 2.832–6.079, p = 0.000), gliomas (HR = 2.650, 95% CI:1.205–5.829, p = 0.015), and SCC (HR = 1.696, 95% CI:1.030–2.793, p = 0.038). However, there was no association between FAK overexpression and survival in colorectal cancer (HR = 1.569, 95% CI:0.981–2.509, p = 0.060), lung cancer (HR = 1.694, 95% CI: 0.719–3.993, p = 0.228), breast cancer (HR = 2.286, 95% CI: 0.423–12.357, p = 0.337), bile duct cancer (HR = 1.995, 95% CI: 0.956–4.164, p = 0.066), and pancreatic cancer (HR = 0.948, 95% CI: 0.587–1.530, p = 0.827). For subgroup analyses based on method, ethnicity, HR estimate method, sample size, and FAK status quality score, all results suggested that FAK overexpression had a poor impact on survival.

**Table 2:** Subgroup analysis of main outcome in vary cancer types.

subgroup	Datasets	pts	HR	95%CI	P for subgroup difference	Heterogeneity	
						I^2^	P value
Overall survival	37	6247	2.073	1.712–2.510		61.2%	0.000
Ethnicity							
Asian	25	4404	2.221	1.823–2.707	0.000	39.9%	0.022
Non-Asian	12	1843	1.803	1.207–2.694	0.004	77.8%	0.000
Method							
protein	34	5463	2.069	1.668–2.566	0.000	61.8%	
Gene	3	784	2.065	1.281–3.326	0.003	66.9%	0.049
HR estimate							
Direct	23	4871	2.174	1.782–2.654	0.000	51.7%	0.002
Indirect	14	1376	1.760	1.127–2.749	0.013	71.5%	0.000
Tumor type							
Gastric	7	2370	2.646	1.743–4.017	0.000	74.9%	0.001
Liver	5	378	1.788	1.228–2.602	0.002	0.0%	0.746
Ovarian	5	547	1.815	1.193–2.762	0.005	0.0%	0.605
Endometrium	2	404	4.149	2.832–6.079	0.000	0.0%	0.725
Gliomas	2	192	2.650	1.205–5.829	0.015	0.0%	0.729
SCC	3	225	1.696	1.030–2.793	0.038	0.0%	0.695
Sample size							
>100	18	4790	2.042	1.606–2.598	0.000	72.9%	0.000
≤100	19	1457	2.137	1.540–2.964	0.000	39.3%	0.041
FAK status							
FAK	30	5126	2.074	1.685–2.553	0.000	56.6%	
pFAK	7	1121	2.108	1.262–3.521	0.004	76.8%	0.000
Quality score							
>3	12	1747	1.947	1.398–2.710	0.000	47.0%	0.031
≤3	25	4500	2.136	1.680–2.717	0.000	67.1%	0.000

Pts, patients;SCC, squamous cell carcinomas.

**Fig 3:**
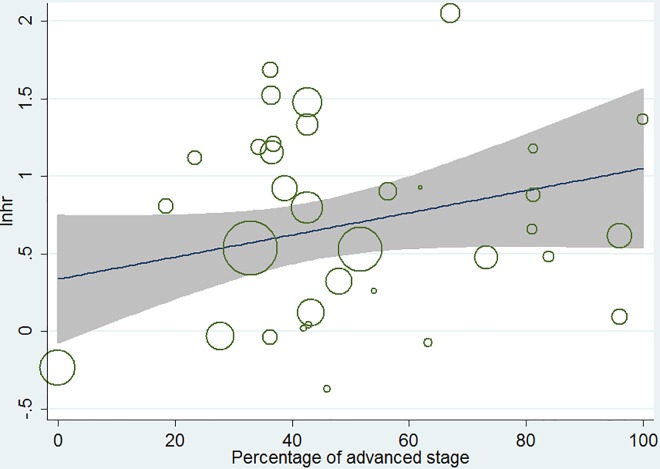
Results of meta-regression. Ln(HR)-ln(percentage of advanced stage).

A meta-regression analysis demonstrated that there was no relationship between HR and the disease stage of patients (p = 0.69, Fig 3).

### Sensitivity analyses

A sensitivity analysis was conducted to test the robustness of the HR estimates by removing studies individually and analyzing the effects on the remaining studies. The result showed that no individual study lay outside the 95% CI of the overall HR estimate.

### Publication bias

**Fig 4:**
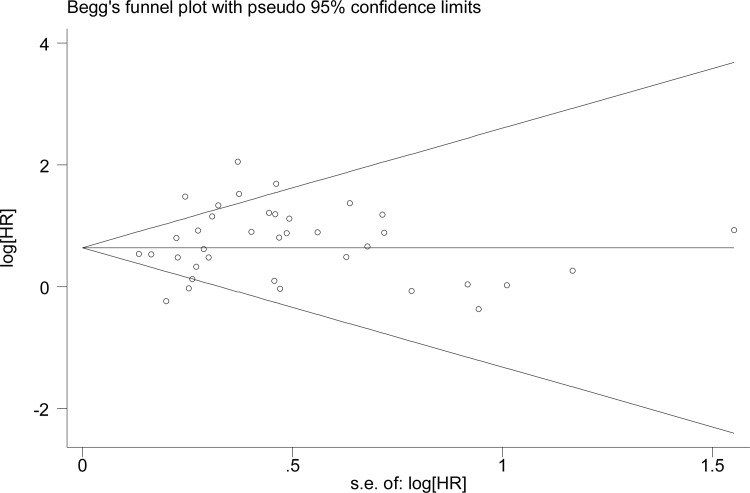
Begg’s funnel plot of FAK expression and overall survival (OS) in patients with solid tumor.

In terms of publication bias estimation, we observed symmetry according to the Begg’s test in all analyses (p = 1.000) (Fig 4), indicating no evidence of small study effects.

## Discussion

FAK is a non-receptor tyrosine kinase that localizes to contact sites in focal adhesions, and holds a key position in the signal transduction network between cells and the extracellular matrix [1–3,59]. Over the years, numerous studies have shown a pivotal role of FAK in tumorigenesis and metastasis. In the present study, we observed that FAK expression was elevated in a broad range of somatic cancers, including astrocytic, breast, cervical, colorectal, endometrial, esophageal, gastric, head and neck, hepatocellular, laryngeal, lung, ovarian, pancreatic, prostate, lung, brain, skin, and thyroid cancers [8,15,16,30,60–70]. Furthermore, FAK overexpression was associated with aggressive human cancers. FAK can promote cancer invasion and metastasis [71]. In addition, this study confirmed that FAK activation, as determined by phosphospecific antibody recognition of the FAK tyrosine autophosphorylation site, increased with tumor progression. FAK is phosphorylated in response to clustering of integrins, cell spreading, or formation of focal adhesions [1]. At least six tyrosine residues (Y397, Y407, Y576, Y577, Y861, and Y925) have been identified as phosphorylation sites. IHC staining of activated (phosphorylated) receptors may be more informative than immunostaining of single markers regardless of their activation status. These above results suggest that high expression of both FAK and phosphorylation status may be a target for cancer therapeutics and may have an impact on survival.

A number of studies have shown that high FAK expression and activity are associated with not only malignancy [72,73], but also with poor prognosis [47]. FAK overexpression was positively correlated with lymph node and distal metastasis, as well as with a significant reduction in patient OS[12,47,48,74]. In this study, the primary results confirm that FAK expression is associated with poor prognosis based on pooled HR estimates. FAK was associated with worse OS in gastric cancer, hepatocellular carcinoma, ovarian cancer, endometrial cancer, gliomas, and squamous cell carcinoma. Interestingly, we also found overexpression of FAK was not associated with worse outcome in some types of solid cancers. This discrepancy may be partly explained by the small sample size in the individual studies. Although Begg’s test showed no evidence of publication bias and while sensitivity analyses further supported the robustness of the meta-analysis findings, it is essential for larger studies to enroll patients with specific tumor types in future studies. Besides FAK expression, its activation plays a critical role in tumor progression and prognosis. In some types of cancers, such as colorectal cancer, although the total expression of FAK was reported not to be associated with survival [14], the phosphorylation status was demonstrated to have an impact. In human colorectal cancer, nuclear expression of phosphorylated FAK is associated with poor prognosis. Albasri et al. [54] reported positive nuclear P-FAK expression was associated with shorter disease-specific survival in univariate (p = 0.005) and multivariate analysis (p = 0.016). In breast cancer, it was reported that increased FAK activity frequently correlates with metastatic disease and poor prognosis [75]. Differences in technique, IHC staining antibody and cut off values for positive protein expression may also have accounted for the observed heterogeneity. Overall, these results suggest that FAK may be an important marker for poor prognosis in a group of solid tumor patients.

The meta-regression analysis revealed no association between the overall HR and the percentage of advanced stage patients. This result supported that the prognostic value of FAK was independent from disease stage in solid tumors.

There are some limitations to this meta-analysis. First, the quality of individual studies was not always optimal. Second, the approach of extracting HRs from K-M curves could be a potential source of heterogeneity. Conversion of K-M curves could misestimate the variance of HRs, although the subgroup analysis did not indicate any major deviation. Thirdly, after cancer types were stratified, the sample size included in the meta-analysis was relatively small. The Gene Expression Omnibus(GEO) database includes a massive amount of gene chip data that profiles gene expression in many tumor types. The inclusion of GEO data would likely result in a more extensive data source and more realistic results. In the future, we will conduct a new study focusing on the GEO database.

In conclusion, through combining different study results, our meta-analysis provides evidence that FAK is associated with worse OS in diverse solid tumor types. In addition, high-quality studies should also be carried out to identify the potential role of FAK expression and phosphorylation status in solid tumors for clinical prognosis and treatment decision making.

## Supporting Information

S1 Checklist The section that contains each item in PRISMA Checklist. (DOC)

S1 Table Evaluation of FAK in the selected studies. (DOC)
