# Supplementary material for: Prognostic Value of Focal Adhesion Kinase (FAK) in Human Solid Carcinomas: A Meta-Analysis
Source: PLoS One. 2016 Sep 16;11(9):e0162666. doi: 10.1371/journal.pone.0162666 (PMC5026375; doi:10.1371/journal.pone.0162666)
Supplement: S1 Table — (DOC) [file pone.0162666.s002.doc]

S1 Table. Evaluation of FAK in the selected studies.

| Ref | FAK status | Antibody(clone) | Cut off for overexpression |
| --- | --- | --- | --- |
| Albasri 2014 | pFAK | Anti-P-FAK(Y397) | Positive: H-score>30, H score was obtained by multiplying SI and percentage of stained cells. SI was quantified from 0 to 3(0= no staining, 1=weak staining, 2=moderate and 3=strong staining) |
| Dy 2014 | FAK | FAK4.47 primary  antibody | Positive: tumor score≥3, score=SI(0,none;1+,weak; 2+,moderate; 3+, strong) plus extent of staining(extent 0, no staining in three cores; 1, only one core had a positive staining, mild; 2, only two cores had a positive staining, moderate; 3, all three cores had the positive staining,diffuse). |
| Zhang 2014 | FAK | Mouse anti-FAK | High: score≥4, SI (0,none; 1, weak,;2, moderate; 3, strong) and staining percentage(0,≤5% positive cells,;1,6-25% positive cells;2,26-50% positive cells; 3,>51% positive cells) were used for evaluation. |
| Gao 2014 | FAK | PCR | Segregated into high/low expression groups based on ROC analysis |
| Chen 2013  (training) | FAK | Monoclonal rabbit  anti-FAK | SI (0,none; 1, weak,;2, moderate; 3, strong) and staining percentage(1,0-25%; 2,26-50%; 3,51-75% ;4, 76-100%) were used for IRS. Cutoff value was obtained by ROC analysis. |
| Chen 2013  (validation) | FAK | Monoclonal rabbit  anti-FAK | SI (0,none; 1, weak,;2, moderate; 3, strong) and staining percentage(1,0-25%; 2,26-50%; 3,51-75% ;4, 76-100%) were used for IRS. Cutoff value was obtained by ROC analysis. |
| Ji 2013 | FAK | Mouse anti-human FAK 4.47 primary  monoclonal antibody | High: moderate staining in ≥80% of tumor cells or strong staining in ≥25% of tumor cells |
| Zhou 2013  (FAK) | FAK | NR | The staining was scored based on intensity(0-3+) and distribution(<10%=1, 10-50%=2,>50%=3) and a staining index was calculated. Staining>2 was scored as positive. Cytoplasmic staining alone for FAK and pFAK≥2 was scored as positive. |
| Zhou 2013  (pFAK) | pFAK | NR | The staining was scored based on intensity(0-3+) and distribution(<10%=1, 10-50%=2,>50%=3) and a staining index was calculated. Staining>2 was scored as positive. Cytoplasmic staining alone for FAK and pFAK≥2 was scored as positive. |
| Garouniatis 2013 | pFAK | Rabbit anti-FAK  (p125-FAK) | FAK expression evaluation was based on a scoring system that measured intensity(0 for none, 1 for borderline, 2 for weak, 3 for moderate and 4 for strong) and percentage of positive cells (over or below 50%). |
| Kim 2012 | FAK | Polyclonal anti-FAK | SI was determined as negative, 0; weak,1; moderate, 2; and strong 3. Stained area was divided into four groups:negative, 0;≤25% of cells, 1; >25% and ≤50% of cells, 2; >50% of cells,3. ≥3 was defined as positive immunoreactivity. |
| Qayyum 2012 | pFAK | Rabbit anti-pFAKY861 | High: above median histoscore. The weighted histoscore grades staining intensity as negative(0), weak(1), moderate(2) and strong(3), then multiplication of the percentage of tumor cells within each category. |
| Theocharis 2012 | FAK | Mouse anti-human FAK IgG1 antibody | High:score≥3. Score was according to the percentage of positive tumor cells as 0: 5-24% of tumor cells positive; 2:25-49%; 3:50-100%, and its intensity as 0:negative staining,1:mild staining;2:intermediate staining;3: intense staining. |
| Fan 2011 | pFAK | Mouse monoclonal  antibody against human p-FAK Y397 | High: score≥3. score was obtained by multiplying SI(0:negative staining;1:weak;2:moderate;3:strong) and pencentage of stained cells(0:<20% positive staining;1:20-80%;2:>80%). |
| Yom 2011  (FISH) | FAK | FISH | Positive: high polysomy(≥4 copies in ≥40% of cells and gene amplification(chromosome ratio of ≥2 or ≥15 copies of FAK per cell in ≥10% of analyzed cells). |
| Yom 2011  (IHC) | FAK | FAK monoclonal  antibody 4.47 | Overexpression: 20% or more carcinoma cells stained with intensity score 3+. |
| Park 2010  (IHC cyto) | FAK  (cyto) | Anti-FAK monoclonal antibody | Positive: focal(labeling in 10%-% tumor cells) and diffuse(labeling in >50%) immunoreactivity |
| Park 2010  (IHC mem) | FAK  (mem) | Anti-FAK monoclonal antibody | Positive: focal(labeling in 10%-% tumor cells) and diffuse(labeling in >50%) immunoreactivity |
| Park 2010  (FISH) | FAK | FISH | Positive: high-level amplification(FAK/CEP8 ratio great than or equal to 2 in more than 10% of tumor cells. |
| Yuan 2010 | FAK | H-1 antibody | Positive: weak(cytoplasm of tumor cells showed yellow or brown and the percentage of stained tumor cells out of all tumor cells was between 25%(including 25%)) and strong positivity(cytoplasm of tumor cells showed yellow or brown and the percentage of stained tumor cells out of all tumor cells≥75%) |
| Chatzizacharias 2010 | FAK | Mouse anti-human FAK IgG1 antibody | High: higher than the median value(35% for FAK) |
| Ding 2010  (FAK) | FAK | Primary antibodies  against FAK  (rabbit polyclonal IgG) | Samples were scored as follows based on the grades of both percentage and intensity: negative(-)(score 0 to 1), low to moderate positive (+) (score 2 to 3), and strong positive(++)(score>3). The percentage of positively stained cells:0,positively stainedcells<25%; 1,25% to 75%;and 2, 75%. SI: 0, weak yellow; 1, yellow, and 2, dark yellow or brown. Negative (−) (score 0 to 1) and positive (+) (score≥2) |
| Ding 2010  (pFAK) | pFAK | Phosphor-FAK(Tyr397)(rabbit polyclonal IgG) | Samples were scored as follows based on the grades of both percentage and intensity: negative(-)(score 0 to 1), low to moderate positive (+) (score 2 to 3), and strong positive(++)(score>3). The percentage of positively stained cells:0,positively stainedcells<25%; 1,25% to 75%;and 2, 75%. SI: 0, weak yellow; 1, yellow, and 2, dark yellow or brown. Negative (−) (score 0 to 1) and positive (+) (score≥2) |
| Hayashi 2010 | FAK | Mouse monoclonal  antibody; clone 4.46) | Positive: positive cells >33% |
| Giaginis 2009 | FAK | Mouse anti-human FAK antibody | Positive: more than the mean percentage value of tumoral cells. |
| Wang 2009 | FAK | NR | Positive: presence of immunoreactivity in at least 5% of cancer cells. |
| Sun 2007 | FAK | Primary antibodies | High: over 50% of tumour section |
| Furuyama 2006 | FAK | Mouse antihuman FAK antibody(05-537) | Positive: score greater than 2. Score was multiplied by SI(0,none; 1,borderline;2, weak;3,moderate;4,strong) and proportion of positively stained cells among cancer cells(0=none;1=1-49%;2=50-100%). |
| Ohta 2006 | FAK | Mouse monoclonal  antibody | High: >10% positive cells |
| Sood 2004 | FAK | Mouse anti-human  FAK , clone 4.47 | High: moderate(OS=2,3 to 4 points) and strong expression(OS=3, 5 to 6 points). Points for expression(1 poits, weak intensity; 2 points, moderate intensity; 3 points, strong intensity) and percentage of positive cells(o points,0 to 5%;2 points, moderate intensity; 3 points, >50%) were added and an OS was assigned. |
| Itoh 2004 | FAK | Mouse monoclonal  antibody; clone 4.47 | Positive: >20% of the carcinoma cells were stained positive for protein |
| Miyazaki 2003 | FAK | Monoclonal antibody  specific for FAK  (clone 4.47) | Overexpression: >40% of carcinoma cells were stained more intensely than the normal epithelial basement membrane. |
| Jan 2009 | FAK | Anti-FAK(C-20) antibodies | Overexpression: Q score greater than 2. Q score was caculated by summing intensity(0:negative; 1: weak; 2:moderate; 3:strong) and heterogeneity scores(proportion of positive staining of tumor cells, 0,0%;1,1% to25%;2,26% to 50%；3,51% to 75%; 4, 76% to 100%) |
| Li 2012 | FAK | Rabbit anti focal  adhesion kinase  polycolonal antibody | NR |
| Aust 2014  (FAK) | FAK | Primary antibody  (monoclonal mouse  IgG1) | High:2-3,moderate to high expression. Scoring system was based on staining intensity(0-3) and percentage of positive cells |
| Aust 2014  (pFAK) | pFAK | Primary antibody  (polyclonal rabbit) | High: 2-3,moderate to high expression. Scoring system included staining intensity and percentage of postitive cells. |
| Li 2015 | FAK | Rabbit anti-human  polyclonal antibody | Negative: cells with brown granules in the cytoplasm and/or membrane. |

ROC, receiver operating characteristics; IRS, immunoreactivity score; OS, overall score; RT qPCR, reverse transcriptase quantitative polymerase chain reaction; IHC, immunohistochemistry; FISH, fluorescence in situ hybridization; NR, not reported
